# Evaluation of electroporated area using 2,3,5-triphenyltetrazolium chloride in a potato model

**DOI:** 10.1038/s41598-021-99987-2

**Published:** 2021-10-14

**Authors:** Seung Jeong, Hongbae Kim, Junhyung Park, Ki Woo Kim, Sung Bo Sim, Jong Hoon Chung

**Affiliations:** 1 Department of Biosystems and Biomaterials Science and Engineering, Seoul National University, Seoul, 08826 Republic of Korea; 2 Department of Ecology and Environmental System, Kyungpook National University, Sangju, 37224 Republic of Korea; 3 Department of Thoracic and Cardiovascular Surgery, The Catholic University of Korea, Bucheon, 14647 Republic of Korea; 4 Research Institute for Agriculture and Life Sciences, Seoul National University, Seoul, 08826 Republic of Korea

**Keywords:** Biophysical chemistry, Membrane biophysics, Biochemistry

## Abstract

Irreversible electroporation (IRE) is a tissue ablation method, uses short high electric pulses and results in cell death in target tissue by irreversibly permeabilizing the cell membrane. Potato is commonly used as a tissue model for electroporation experiments. The blackened area that forms 12 h after electric pulsing is regarded as an IRE-ablated area caused by melanin accumulation. Here, the 2,3,5-triphenyltetrazolium chloride (TTC) was used as a dye to assess the IRE-ablated area 3 h after potato model ablation. Comparison between the blackened area and TTC-unstained white area in various voltage conditions showed that TTC staining well delineated the IRE-ablated area. Moreover, whether the ablated area was consistent over time and at different staining times was investigated. In addition, the presumed reversible electroporation (RE) area was formed surrounding the IRE-ablated area. Overall, TTC staining can provide a more rapid and accurate electroporated area evaluation.

## Introduction

Irreversible electroporation (IRE), a tissue ablation method, uses short high electric pulses that cause low thermal damage and induce apoptotic cell death by irreversibly permeabilizing the cell membrane in the targeted tissue^1^. IRE was recently investigated as an alternative ablation method to several conventional tissue ablation techniques, such as cryoablation, radio-frequency ablation, and microwave ablation^2–5^. The advantages of IRE include producing a sharp boundary between treated and untreated areas, sparing vasculature and ductal networks, which results in more rapid tissue regeneration^6–8^.

Many researchers used potatoes as models to evaluate IRE-ablated areas due to their low cost, relative convenience, and lack of ethical issues^9–12^. The evaluation is conventionally performed by calculating the blackened potato tissue area 12 h posttreatment^13^. The intracellular polyphenol oxidation results in melanin accumulation that forms the dark area^14^. However, when using this method, obtaining the posttreatment results requires waiting for long hours. Furthermore, the results are affected by the inhomogeneity of the potato tissue^15^.

Magnetic resonance imaging (MRI) was used for instant imaging of potato tissue changes after pulsing to overcome these disadvantages^16^. The ablation area detected using MRI was similar to the conventional melanin pigmentation ablation outcome in various electric field strengths. However, using MRI for most researchers is unsuitable due to its high cost and uneasiness to handle.

The blue dye was used to evaluate IRE-ablated areas on the potatoes using the osmotic equilibrium phenomenon, but RE areas were not detected ^17^.

The water-soluble tetrazolium salt has been used to evaluate cell viability in in vitro and ex vivo experiments^18–21^. The 2,3,5-triphenyltetrazolium chloride (TTC) is a representative agent for this purpose and is reduced to insoluble red-colored triphenylformazan crystals by the dehydrogenase produced by mitochondria in cells^22^. The IRE-ablated area was evaluated with TTC in ex vivo swine pancreatic tissue^20^. The TTC-unstained white area was an IRE-ablated area in the swine pancreas tissue. This type of TTC staining has a rapid chemical reaction and reduces evaluation time. However, research is needed to evaluate the IRE-ablated area potato models using TTC staining.

Here, an IRE-ablated area was assessed based on TTC staining in a potato model after pulsing. The TTC-unstained white area was compared with the blackened area by the conventional melanin accumulation method. Moreover, the consistency of the TTC-unstained white area was examined to verify its accuracy and usefulness in IRE research. Moreover, the presumed-RE area was formed with the TTC staining after IRE pulsing on the potato. Furthermore, the correlation between the ablation area and the electrical properties of potatoes was examined to evaluate the suitability of the TTC staining method for electroporation research.

## Materials and methods

### Electric fields simulation

The geometry of the model was adopted from a previous study^23^. The electric field strength between electrodes is governed by the Poisson equation as follows:

1 $$\nabla^{2} \emptyset = - \frac{{\uprho }}{{{\upvarepsilon }_{0} }}$$

If the conductivity of the potato is supposed to be constant, a steady-state electric field distribution was obtained by solving the equation as follows:

2 $$\nabla^{{2}} \emptyset = 0$$

where $\emptyset$ is the electric field strength, assuming the electrode length is larger than the distance between electrodes. The surface effects of the potato are neglected. The electric field strength is presented based on a gradient of the electric potential:

3 $$E = \nabla \emptyset$$

The boundary condition is defined as either ∅ = *V*_0_ or 0 in the applied tissue. The resting boundaries were assumed as electrically insulating ($\frac{\mathrm{d\varnothing }}{dn}$ = 0). The electric field was estimated using the EPO code™ developed with the OpenFOAM (The Standard Co. Ltd., Gunpo-si, Republic of Korea).

### Potato tissue ablation

**Figure 1:**
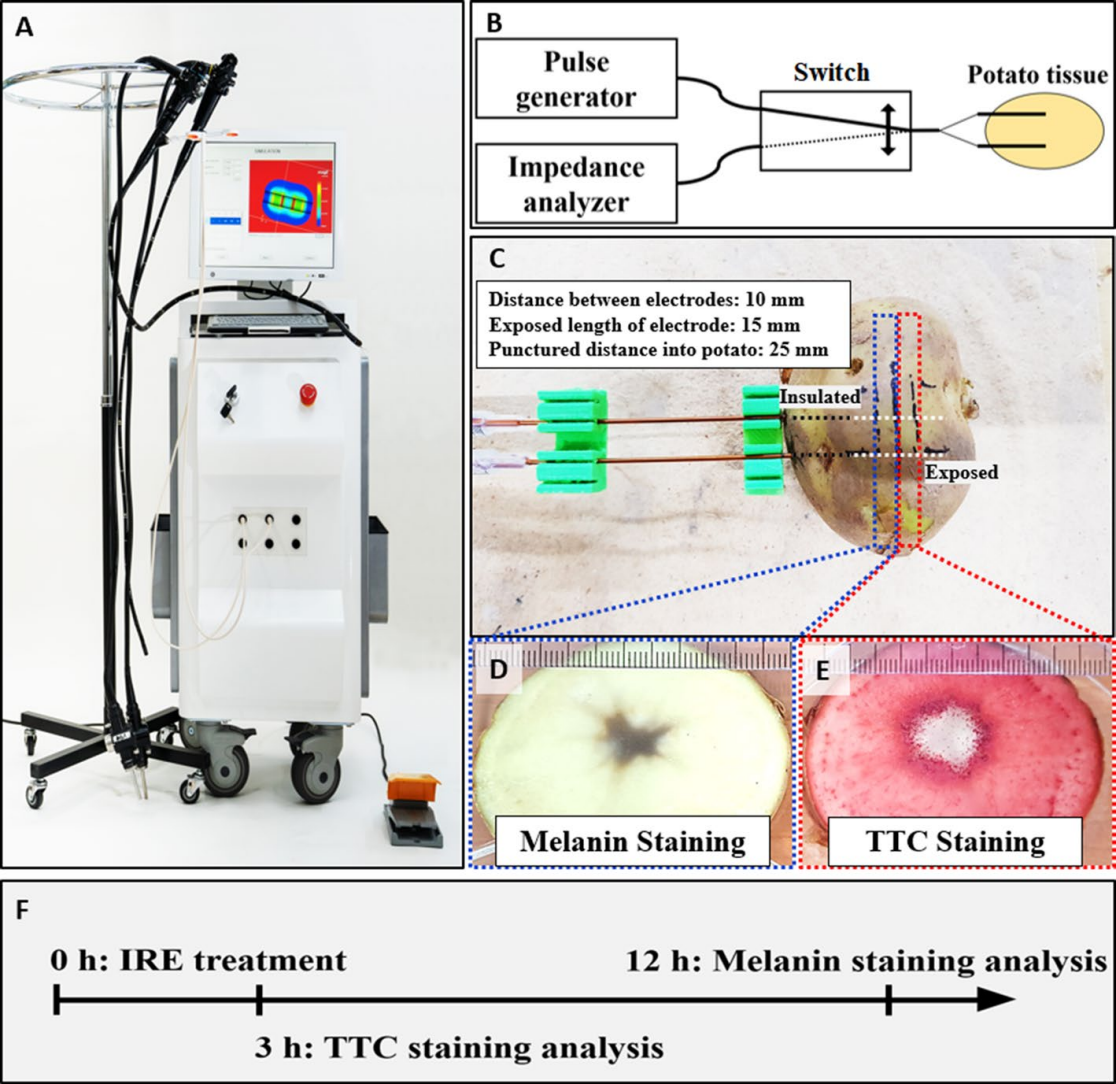
Experimental setup for comparing melanin accumulation and TTC staining methods. (**A**) The EPO-S1 system (The Standard Co., Ltd.) was used as a pulse generator for IRE. (**B**) An impedance analyzer with a relay was used to measure the conductivity between the electrodes in the potato. (**C**) The electrodes had a 1-mm outer diameter, 10-mm distance, 10-mm exposure (not insulated part), and 25-mm puncture distance into the potato. The potato was punctured with electrodes. (**D**) The left side of the sectioned potato was accumulated with melanin from the middle point of the exposed needle. (**E**) The right side was stained with TTC. (**F**) The experimental timeline shows TTC staining analysis was faster than the melanin accumulation analysis.

A single batch of large-size (weight, ~ 240 g; length, 80 mm; and width, 60 mm) potato tubers from the same harvest were purchased from a local grocery store (Gunpo-si, Republic of Korea). All experiments were performed using the EPO-S1 generator (The Standard Co., Ltd.), a prototype pulse generator for IRE (Fig. 1A). Two 6-Fr-diameter stainless-steel needle electrodes with a 15-mm exposure length, inserted in parallel, normal to the potato slice surface, with a 10-mm center-to-center distance between the electrodes (Fig. 1C), delivered the electric pulses. The treatment consisted of a square-pulsed electric field with 300, 600, 900, 1200, or 1500 V/cm, 100 μs pulse width; 2000 μs pulse delay; and 32 pulse numbers to cause a sufficient IRE-ablated area according to the pre-test (Supplementary Tables 1, 2). In the different staining time experiments, electric field strength 1000 V/cm, 100 μs pulse width, 2000 μs pulse delay, and 100 pulse numbers were used with the same electrodes as described above.

### Conductivity and current measurement

The conductivity measurement was performed using 4192A LF Impedance Analyzer (Yokogawa-Hewlett-Packard Ltd., Japan). A manual switch was placed between the experimental object and the analyzer for measurements after pulsing (Fig. 1B). The conductivity was measured before pulsing, and the switch was turned off to ensure a connection between the object and pulse generator. After pulsing, the switch was immediately turned on to connect the analyzer to the object and was manually operated. The conductivity was measured using a 10-Hz frequency. A digital oscilloscope (TDA3044B, Tektronix, USA) was used for current measurements. A hole-type current probe (TCP305A, Tektronix) was clamped to a code connecting the pulse generator and the electrode.

### Melanin accumulation and TTC staining methods

**Figure 2:**
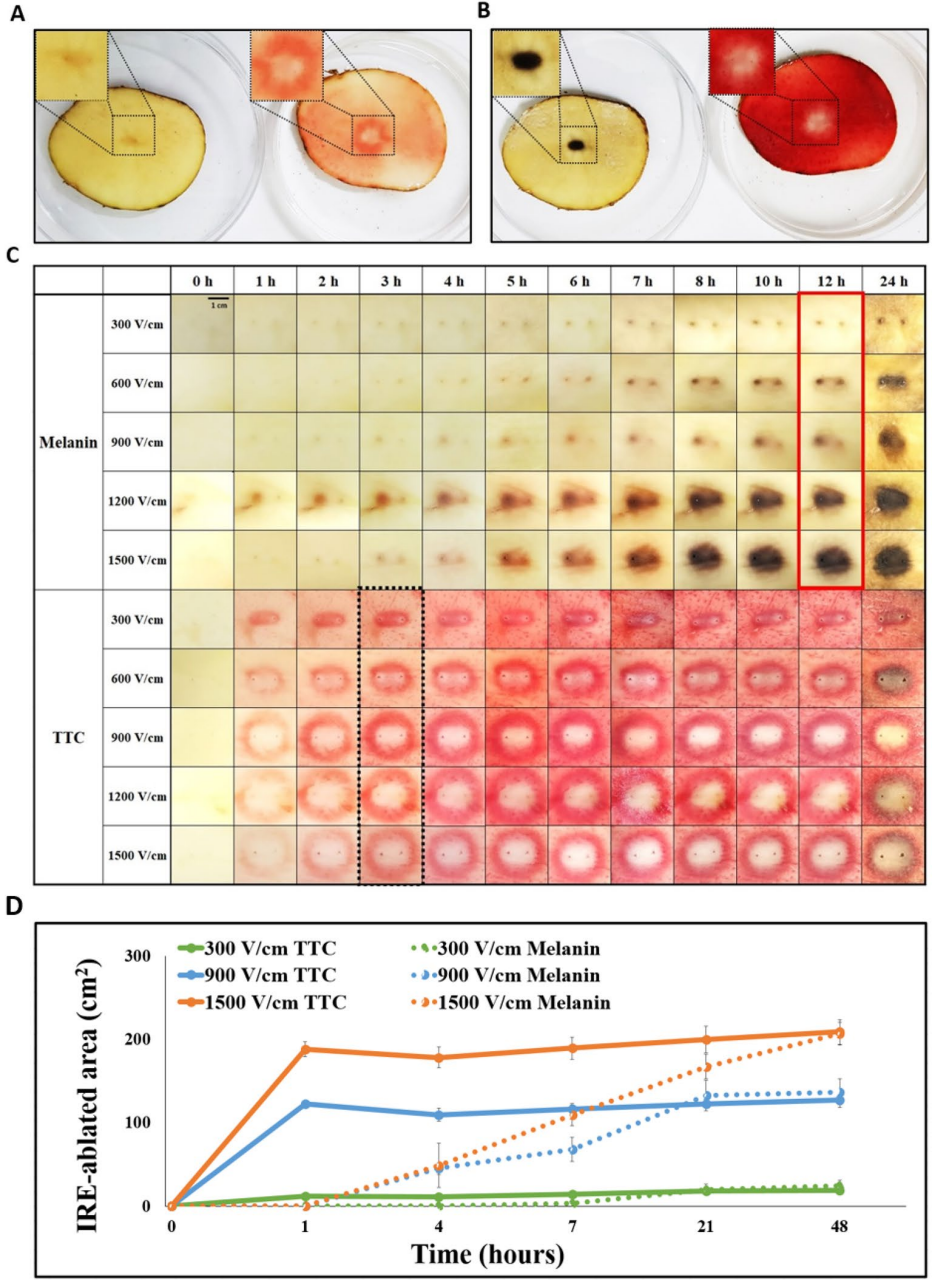
Comparison of melanin accumulation and TTC staining over time. (**A**) Three hours after pulsing, TTC staining showed a distinct IRE-ablated area (right) compared with melanin accumulation, which showed a minimal tissue change (left). (**B**) Twelve hours after pulsing, the samples for melanin accumulation also showed a distinct IRE-ablated area (left), and the white dead cell area remained in TTC staining (right). (**C**) Melanin accumulation and TTC staining in the treated potato during 24 h at various voltage conditions. The samples for melanin accumulation showed the ablated area 12 h after pulsing, and TTC staining showed the clear demarcation line of the ablated area 3 h after pulsing. (**D**) IRE-ablated area comparison graph between melanin accumulation and TTC staining during 48-h period.

A section from the treated potato was cut in 5 mm (width) × 4–6 cm (length) and placed at room temperature in about 60% humidified environment for 48 h for melanin accumulation (Fig. 1D). Another section was submerged into a dish containing 20 mL of 0.5% w/v TTC (Kanto Chemical Co., Ltd., Japan) in normal saline for 48 h (Fig. 1E). The results were quantitatively compared after 3 h of TTC staining and 12 h of melanin accumulation (Fig. 1F). TTC staining was performed within 5 min after pulsing. Photographs were taken every hour for 8 h, and then once at 10, 12, and 24 h thereafter. Melanin accumulation and TTC staining images were compared (Fig. 2C). ImageJ 1.53c (Wayne Ribband, National Institutes of Health, USA) software was used to evaluate the IRE-ablated area in the potato models. The distance was set using a ruler in the software. Trainable Weka Segmentation was performed to create a binary image of the white area and deep red area caused by TTC staining to measure the IRE and the presumed reversible electroporation (RE) areas. Moreoer, areas with accumulated black melanin were calculated using the same method.

### Transmission electron microscopy (TEM)

Fragments (1 × 3 mm) were excised from pulsed, inner medullar zones (IRE and deep red areas), and non-pulsed, perimedullar zones of the pulsed potato tuber. They were prefixed in 2% (w/v) paraformaldehyde and 2% (w/v) glutaraldehyde in 0.05 M sodium cacodylate buffer (pH 7.2) at 4 °C overnight. They were washed with cacodylate buffer and post-fixed in 1% (w/v) osmium tetroxide at 4 °C for 2 h. The osmicated specimens were en bloc stained with 1% (w/v) uranyl acetate at 4 °C overnight. The specimens were immersed in a graded ethanol series for dehydration, treated with 100% propylene oxide, and embedded in Spurr’s resin. Ultrathin sections were obtained using an ultramicrotome (Leica UC7; Leica Microsystems, Wetzlar, Germany) and were mounted on 150-mesh copper grids. They were stained with 2% (w/v) uranyl acetate and Reynolds’ lead citrate for 7 min each. The sections were examined using TEM (JEM1400plus; JEOL, Tokyo, Japan) at 120 kV.

### Statistical analysis

Experimental results were expressed as mean ± standard deviation from three repetitions. A two-tailed paired Student’s *t* test was conducted to determine the significance of differences in means (*P < 0.05; **P < 0.01; ***P < 0.001). Pearson’s correlation statistics was used to determine correlation between electrical properties and ablated area of the two different staining methods after IRE. Statistical analysis was performed using Microsoft Excel 2013 (Microsoft Co., Ltd., Redmond, WA, USA) software.

## Results

### Melanin accumulation and TTC staining over time

Based on the experimental scheme (Fig. 1), two staining methods were compared. Representative melanin accumulation and TTC staining are shown in Fig. 1D,E. Three hours after pulsing, the samples for melanin accumulation only showed slight changes, and a prominent ablation area was not observed. However, the TTC staining showed a clear white ablation area inside the deep red area (Fig. 2A). After 12 h, the ablation region in the melanin accumulation became dark black, and in the TTC staining, the white dead cell area remained (Fig. 2B). The red, color-stained area in the TTC staining became visible 1 h after pulsing and showed a clear demarcation 3 h posttreatment compared with melanin accumulation, which required > 12 h after pulsing for visualization of results (Fig. 2C).

The results showed that IRE-ablated areas by TTC staining showed consistency over various electric field strengths (Fig. 2D). The melanin accumulation area increased over time and was similar to TTC staining areas 48 h posttreatment.

### Comparison of melanin accumulation and TTC staining at various electric field strengths

**Figure 3:**
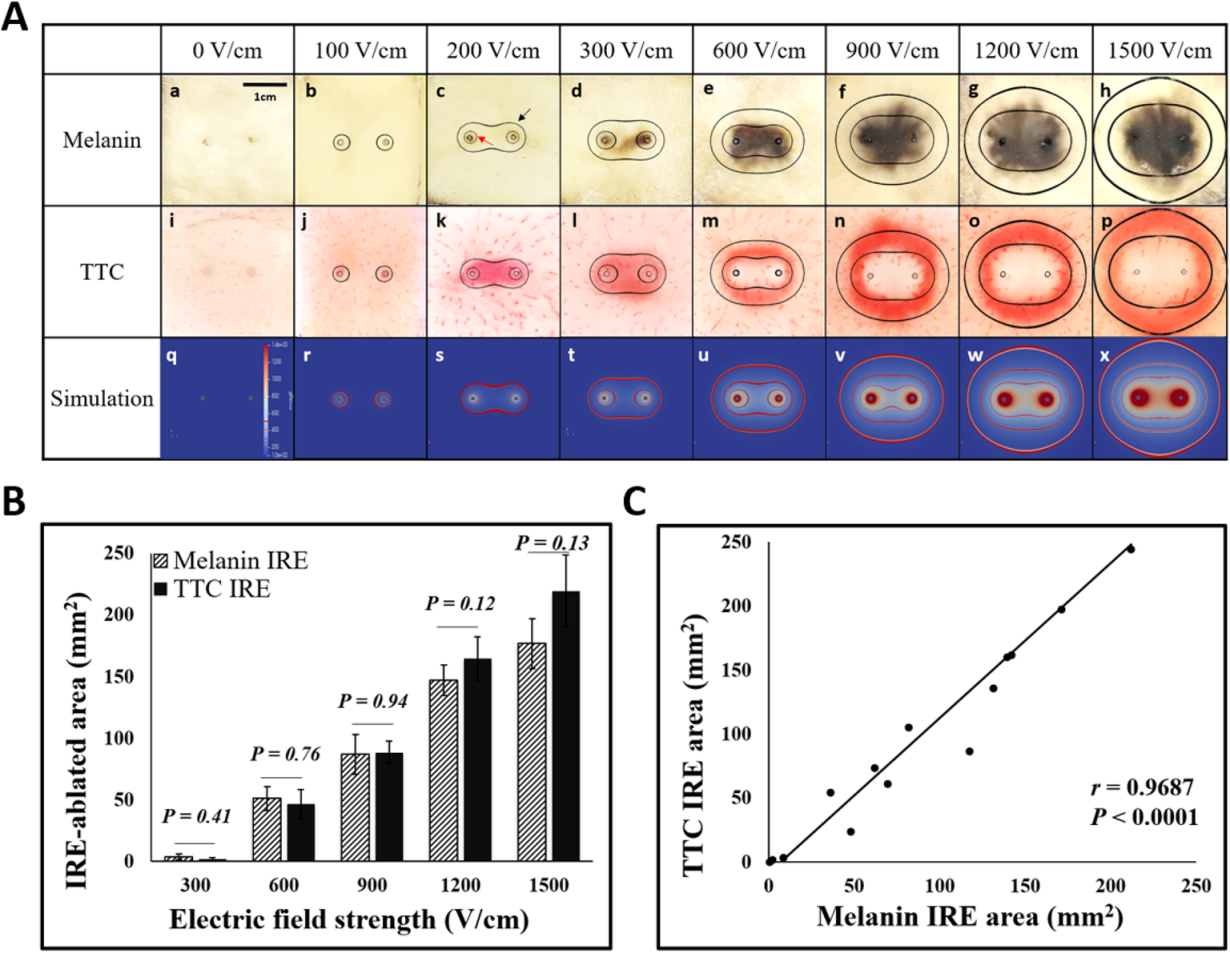
(**A**) Comparing melanin accumulation and TTC staining methods for the IRE-treated area in the potato model at various electric field strengths. (a–h) Conventional melanin accumulation results after pulsing with simulation results (red arrow indicates 250 V/cm line; black arrow indicates 100 V/cm line). (i–p) TTC staining results after pulsing with simulation results. (q–x) Simulation results were calculated using the OpenFOAM program for each condition (red lines indicate 500, 250, and 100 V/cm from the inside). (**B**) Histogram of IRE-ablated area in potato accumulated melanin or stained with TTC. In both staining methods, the ablation area increased depending on electric field strength. (**C**) Correlation data showed the two staining methods for IRE were similar with a high correlation value of 0.9687 (P < 0.0001) using Pearson's correlation statistics. A two-tailed paired Student’s *t*-test was used to determine significance (*P < 0.05; **P < 0.01; ***P < 0.001).

IRE treatment was performed on potatoes with increasing electric field strength to compare the ablation area using melanin accumulation and TTC staining (Fig. 3A). The ablation area significantly increased depending on the intensity of the field strength in both staining methods (Fig. 3B). In general, both the TTC-unstained white area and the blackened area in melanin accumulation seemed similar. Ablated areas in both methods were not significantly different at the various electric field strengths (Fig. 3B, P  > 0.05). With a Pearson’s correlation coeeficient of 0.9687, the ablated areas stained by the two methods were highly correlated (Fig. 3C, P  < 0.0001).

### Current and conductivity changes at the various electric field strengths

**Figure 4:**
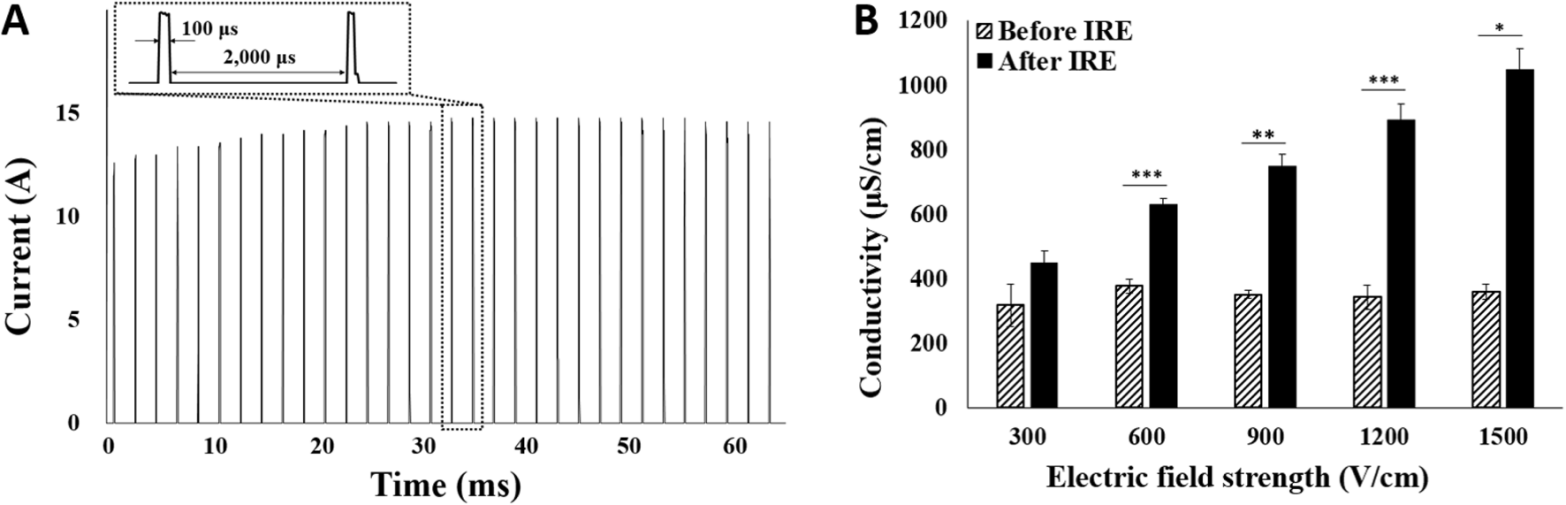
Electrical properties, such as (**A**) current flow after 1500 V/cm 32-pulse stimulation and (**B**) conductivity changes at 10 Hz, before and after pulsing. Conductivity change ratio increased as electric field strength increased. A two-tailed paired Student’s *t*-test was used to determine significance (*P < 0.05; **P < 0.01; ***P < 0.001).

Electrical properties, such as current and conductivity change, were estimated to investigate the correlation between the two staining methods (Fig. 4). Current that passed through the potato tissue after applying 32 pulses of 1500 V/cm is shown in Fig. 4A. The inbox in Fig. 4A shows several square pulses of 100 μs pulse width and 2000 μs interpulse delay. The current gradually increased from 12.5 to ~ 15 A and saturated after the 17th pulse. Conductivity at 10 Hz was measured before and after pulsing for the different electric field strengths. The conductivity tended to increase linearly with the electric field strength (Fig. 4B). A 10-Hz frequency was selected to measure the conductivity because, compared with the other frequencies, it provides the most significant difference in conductivity after pulsing (Supplementary Table 3).

### TTC staining at different times

**Figure 5:**
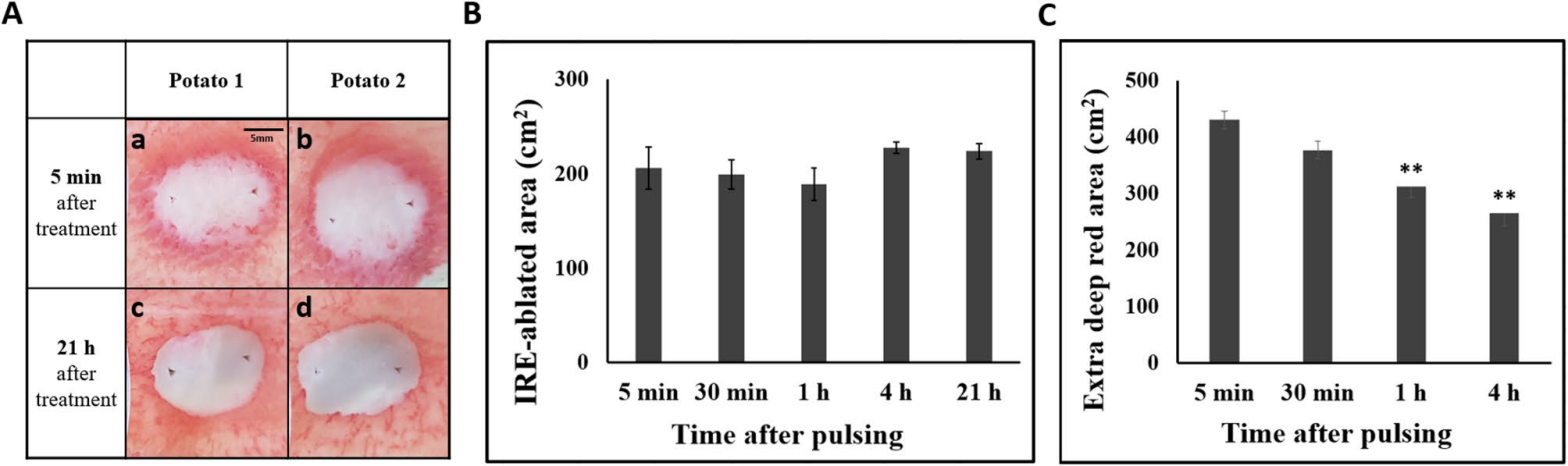
IRE-ablated area at different staining times after pulsing. (**A**) After treating the potato with IRE, TTC staining was performed within 5 min on half of the potato (a, b) and 21 h after pulsing on the other half of the potato (c, d). (**B**) Result comparisons of the TTC-unstained white areas in IRE-treated potatoes at different staining times. (**C**) Deep red areas at different staining times. A two-tailed paired Student’s t-test was used to determine significance compared with the 5 min group (*P < 0.05; **P < 0.01; ***P < 0.001).

TTC staining was performed at 5 min; 30 min; and 1, 4, and 21 h after pulsing to investigate whether the IRE ablation area changes (Fig. 5A). The white IRE-ablated areas were consistent at the different staining times except for a slight increase in the 4 h and the 21 h groups (Fig. 4B). These results showed the IRE-ablated areas were similar regardless of the staining time (Fig. 5B). Deep red areas decreased as the staining time become late, and the areas at 1 h and 4 h after pulsing were significantly lower than that of 5 min (P < 0.01) (Fig. 5C).

### TEM analysis of the potato tissue by TTC staining

**Figure 6:**
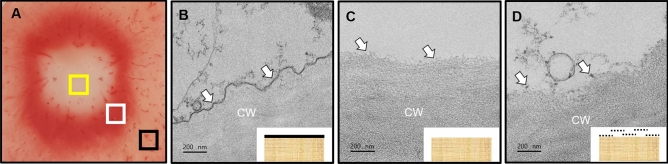
TEM analysis of TTC-unstained and TTC-stained areas after pulsing on the potato tissue. (**A**) The black box indicates a non-pulsed perimedullar zone, the yellow one indicates pulsed TTC-unstained inner medullar zone, and the white one indicates pulsed TTC-stained deep red inner medullar zone. (**B**) Non-pulsed perimedullar zone with evident electron-dense line (arrows) along the cell wall (CW). (**C**) Pulsed TTC-unstained medullar zone with no electron-dense line along with the CW. (**D**) Pulsed TTC-stained deep red inner medullar zone with partially continuous electron-dense lines along with the CW.

TEM revealed whether the continuity of the plasma membrane was preserved after pulsing in the TTC-stained and unstained areas on the potato tissue. Non-pulsed perimedullar zone, pulsed TTC-unstained inner medullar zone, and pulsed TTC-stained deep red inner medullar zone were investigated using TEM (Fig. 6A).

TEM revealed the well-preserved plasma membrane overall attached to the cell wall in the perimedullar zones of the tuber in the non-pulsed perimedullar zone (Fig. 6B, SFig. 2A). Organelles, such as Golgi bodies and mitochondria, were commonly observed in the cytoplasm. The cisternae, lumens, and secretory vesicles could be discerned from the Golgi body (SFig. 2B). Magnified views showed double membranes and cristae of the mitochondria (SFig. 2C). Complex plasmodesmata were often seen between two adjoining parenchyma cells (SFig. 2D).

The cytoplasmic disruption occurred in the IRE inner medullar zone (Fig. 6C, SFig. 3A). The pulsed cytoplasm partially contained electron-dense vesicles with various diameters. The absence of an electron-dense continuous plasma membrane was commonly observed along the cell wall in the magnified views (SFig. 3B). No distinct organelles were found in the cells (SFig. 3C). Magnified views showed the disrupted cytoplasm and plasma membrane detached (to ~ 1 μm) from the cell wall (SFig. 3D).

Partially continuous electron-dense plasma membranes were commonly found in the TTC-stained deep red inner medullar zone (Fig. 6D, SFig. 4A). Magnified views showed the dotted electron-dense remains and detachment of the plasma membrane (to ~ 500 nm) from the cell wall (SFig. 4B). Partially disrupted organelles, including Golgi bodies, were found near the plasma membrane in the cells (SFig. 4C). Small vesicles, ~ 100 nm or less in diameter, were observed between the plasma membrane and cell wall (SFig. 4D). Vesicular bodies appeared to fuse with the plasma membrane.

Magnified views showed differences in the plasma membrane integrity between the inner medullar and perimedullar zones. Continuous electron-dense lines were evident along the cell wall of the perimedullar zones (Fig. 6B). Without a distinct plasma membrane, the fibrillar arrangement was apparent on the cell wall of the IRE inner medullar zones (Fig. 6C). Dotted remains of the plasma membrane were found in the TTC-stained deep red inner medullar zone (Fig. 6D).

## Discussion

Many researchers have evaluated IRE-treated areas in potato models using melanin pigmentation caused by intracellular polyphenol oxidation^9,10,14,24^. This method is convenient to perform because minimal preparation is required. However, > 12 h are required to obtain such results^13^. Furthermore, the blackened area in melanin accumulation may include the RE-treated areas, which are consisted of live cells^25^. Therefore, it is challenging to determine that the blackened area in the treated potato model only consists of dead cells.

TTC staining has several advantages over the currently used melanin accumulation methods regarding evaluating the IRE-treated areas. First, researchers can obtain the ablation results within 3 h after pulsing (Fig. 2A). The recommendation is that experimental time should be short when using potato tissues after pulsing because living tissues, like potatoes, are susceptible to the external environment, such as ambient temperature and humidity. Moreover, the consistency of the IRE-ablated area using TTC staining was verified at different staining times in the present study experiments (Fig. 5B).

Furthermore, TTC staining showed a clear demarcation line within 3 h (Fig. 2C). In TTC staining, the viable cell area becomes red due to the reduction of tetrazolium salt when a living cell remains and the dead cell areas become white^21^. Therefore, TTC staining is more applicable to IRE research^26–28^. TTC-unstained white area is relatively consistent during 48 h, but the melanin accumulation area changes gradually over time (Fig. 2D). At 48 h, the IRE-ablated areas accumulated melanin and finally were similar to the TTC-unstained white areas. These results correspond to the previous study stating that 48 h is the optimal time for melanin accumulation to observe the IRE-ablated area^29^. These TTC staining characteristics provide several advantages for IRE researchers using a potato model, such as more rapid and accurate detection than the conventional method.

Potato tubers were recently adopted to evaluate the ablation area using high-frequency irreversible electroporation (H-FIRE)^29^. Unlike conventional IRE, H-FIRE comprises biphasic pulse trains, making pulse parameter combinations more complicated, such as positive pulse width, negative pulse width, interpulse delay, and inter-phase delay. TTC staining has an advantage when researchers investigate various conditions of H-FIRE due to rapidness and consistency.

TTC staining provides not only IRE-ablated white areas but also deep red areas surrounding white areas (Fig. 3Aj–2Ap). These extra red areas in TTC staining were dyed darker than the outside, which was similar between 100 and 250 V/cm line simulation results (the outermost black line of simulation in Fig. 3Aj–2Ap). The potato cells in these red areas might be alive, referring to the previous study stating that potato cell membranes were intact under a 200 V/cm pulsed electric field^21^. Note that these deep red areas were observed when TTC staining was performed on the potato 5 min, 30 min, 1 h, and 4 h posttreatment (Fig. 5Aa,Ab,4C), whereas the areas were not observed in TTC staining at 21 h after IRE (Fig. 5Ac,4Ad). In general, the cell membrane took a few minutes to reseal after electroporation^30^. The deep red areas decreased as the staining time was delayed, hinting cell membrane resealing was being processed. Based on the TTC staining mechanism, TTC is reduced by dehydrogenase in the cell mitochondria, resulting in a red color formazan^22,31^. Therefore, the color intensity around IRE-ablated areas could be related to the health of the cell. However, it is weird to explain that more cells in the deep red areas were alive because the color was darker than the untreated outer area. Potentially, when the cell membrane is reversibly electroporated with electrical stimulation, TTC molecules are diffused into the cell as per Fick’s law of diffusion^32^. It might trigger more chemical reactions between TTC and dehydrogenase produced in the cell mitochondria, causing a deeper red than in the surrounding area.

Plasma membranes were observed using TEM to ensure these outcomes. Irreversible electroporation was theoretically known to form innumerable nanoscale pores in the plasma membrane^33^. Nevertheless, it is challenging to observe them due to the innate metastable nanopore structures in the bilayer and technical limitations in specimen microscopy preparation^34^. This study demonstrated differences in parenchyma cell ultrastructure between the inner medullar and perimedullar zones of potato tuber (Fig. 6). In the non-pulse potatoes, perimedullar zones showed the overall continuous electron-dense plasma membrane attached to the cell wall (Fig. 6B), and their organelles appeared to have typical structures, such as cisternae and cristae. However, in the pulsed potatoes, IRE inner medullar zone was primarily characterized by the disruption of the plasma membrane and organelles in the cells (Fig. 6D). Instances were noted where partial degradation and detachment of plasma membrane from the cell wall occurred in the presumed-RE inner medullar zone (Fig. D). Such membrane aberrancies have been exemplified using TEM in other organisms^35,36^.

Different membrane disruption levels were noted in the potato tuber between the IRE and presumed-RE inner medullar zones (Fig. 6C,D). The degradation of the plasma membrane was more pronounced in the IRE regions compared with the presumed-RE regions. Given the cellular programs for plasma membrane remodeling, the detachment of the plasma membrane from the cell wall could be assumed to be an intermediate phase for sealing holes^37^. More studies await the elucidation of mechanisms underlying possible plasma membrane sealing and reattachment to the cell wall.

**Figure 7:**
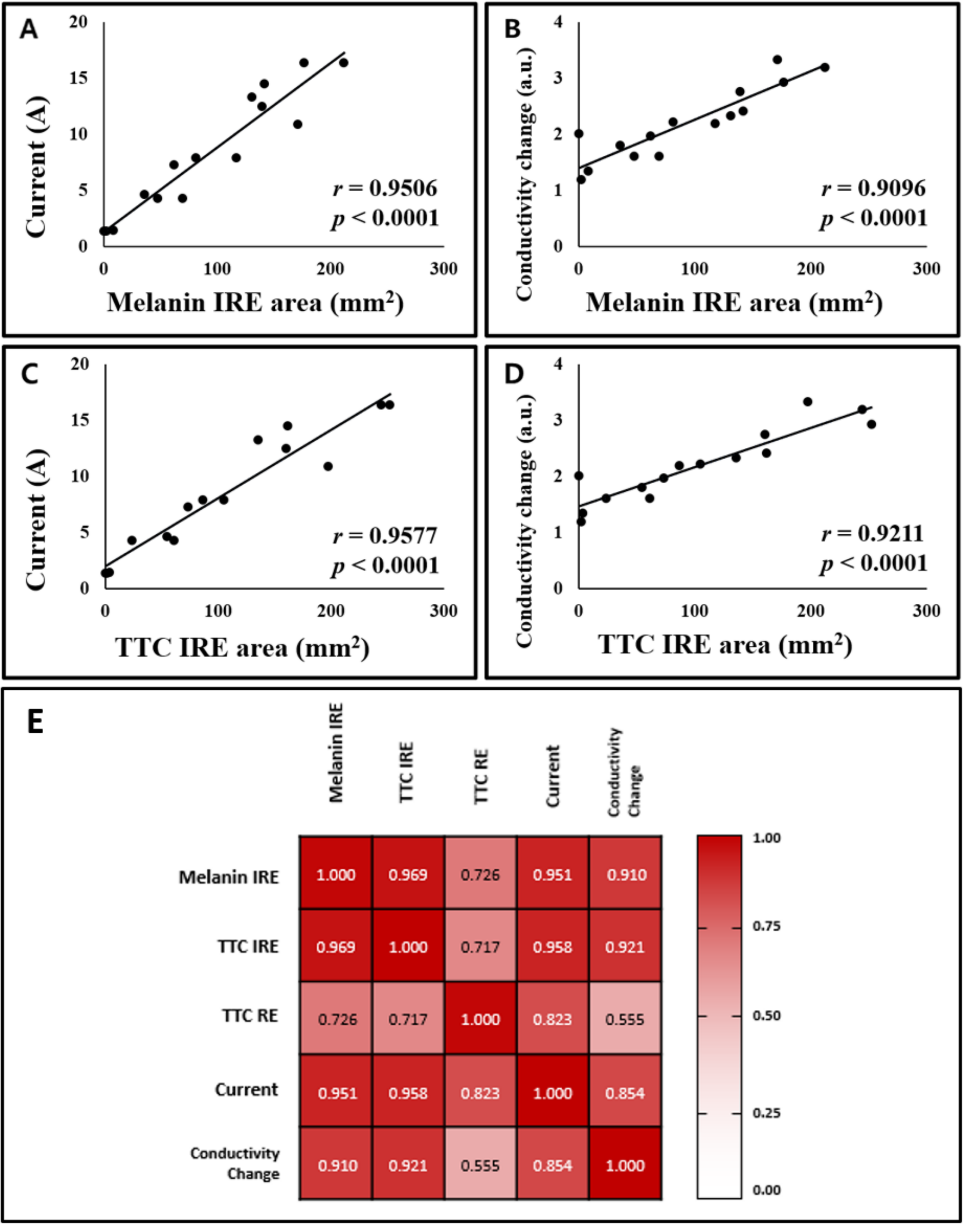
Correlation among electrical properties and each staining method. (**A**) Correlation graph between current and IRE-ablated area by melanin accumulation with a high correlation value of 0.9506 (P < 0.0001). (**B**) Conductivity changes at 10 Hz and IRE-ablated area by melanin accumulation with a value of 0.9096 (P < 0.0001). (**C**) Current and TTC-unstained white IRE-ablated area with a value of 0.9577 (P < 0.0001). (**D**) Conductivity changes at 10 Hz and TTC-unstained white IRE-ablated area with a value of 0.9211 (P < 0.0001). (**E**) Heat map between IRE-ablated area by melanin accumulation, TTC-unstained white IRE-ablated area, TTC-stained presumed-RE area, current, and conductivity changes at 10 Hz. TTC-unstained IRE-ablated area had a higher correlation value than IRE-ablated area by melanin accumulation with current and conductivity change. Pearson’s correlation statistics and P-values were used for correlation and statistical significance.

Furthermore, TTC staining highly correlated with electrical changes in the potato compared with melanin accumulation (Fig. 7). Electrical properties were compared between TTC staining and melanin accumulation; the current that flowed when pulsed electric field stimulation was applied and the conductivity change ratios at 10 Hz represent the extent of cell damage in electrically stimulated tissue^38^. Regarding current, the correlation between current and melanin accumulation area had a correlation coefficient value of 0.9506 (P < 0.0001, Fig. 7A), and between current and TTC-unstained white area had a Pearson’s correlation coefficient value of 0.9577 (P < 0.0001, Fig. 7C). The TTC-unstained white area had a slightly higher correlation with the current than the melanin accumulation area. Regarding the conductivity change ratio at 10 Hz, the correlation between conductivity change ratios and melanin accumulation area had a correlation coefficient value of 0.9096 (P < 0.0001, Fig. 7B), and between conductivity change ratios and TTC-unstained white area had a Pearson’s correlation coefficient of 0.9211 (P < 0.0001, Fig. 7D). The TTC-unstained white area had a slightly higher correlation with conductivity change ratios than the melanin accumulation area. The heat map showed which variables had highly correlated coefficient values (Fig. 7E). Moreover, the TTC-stained presumed-RE area was positively correlated with other values, except for the conductivity change. These relatively low correlations between presumed-RE area and other variables were estimated to be so due to the large measurement deviations, because the delineations of the deep red areas were relatively less pronounced compared with the TTC-unstained white area. Other variables had a correlation coefficient > 0.85. These results showed that TTC staining is an acceptable method for analyzing the IRE-ablated area associated with electrical current and conductivity changes in potato tissue.

**Figure 8:**
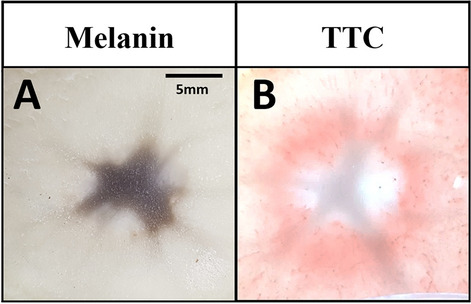
The extent to which the IRE-ablated area is affected by the inner tissue inside the potato. (**A**) Melanin accumulation area after pulsing was star-shaped, affected by the inner tissue. (**B**) TTC-unstained white IRE-ablated area was less affected by the inner tissue and showed an oval shape.

TTC staining showed more consistent results compared with melanin accumulation in potatoes. Homogeneity of tissue is essential to evaluate the IRE-ablated area. However, potato tissue is not very homogeneous, and the ablation outcome can be affected by inner tissues^15^. Here, melanin accumulation area was affected by the inner medullar zone with a star-shaped ablation area. Nonetheless, TTC-unstained white area was less affected by the potato inner medullar zone on other sections of the same potato (Fig. 8). This result indicated that TTC staining could provide a more accurate ablation area when using potato tissues for IRE researches.

The limitation of this study was that the TTC-stained presumed-RE area was not directly identified. Additional microscopy studies are needed to investigate whether the potato cells in the presumed-RE region show reversible nanopores after pulsing. Furthermore, the mechanism of this phenomenon should be investigated in future studies.

In summary, the IRE-ablated area was distinguished with TTC staining in a potato model within 3 h and showed results similar to conventional melanin accumulation area after pulsing. The TTC-unstained white areas were consistent over time and at different staining times. The presumed-RE area was formed with TTC staining in a potato model. The areas were highly correlated with electrical variables such as current and conductivity changes. Hence, TTC staining might be the best choice to evaluate IRE-ablated and RE areas in a potato model.

## Supplementary Information

Supplementary Information.
