# Supplementary material for: Evaluation of electroporated area using 2,3,5-triphenyltetrazolium chloride in a potato model
Source: Sci Rep. 2021 Oct 14;11:20431. doi: 10.1038/s41598-021-99987-2 (PMC8516888; doi:10.1038/s41598-021-99987-2)

Supplementary Table 1. TTC staining on potato tissue 3 h posttreatment at various pulse voltage and pulses with 100 μs pulse width, 200 μs pulse delay


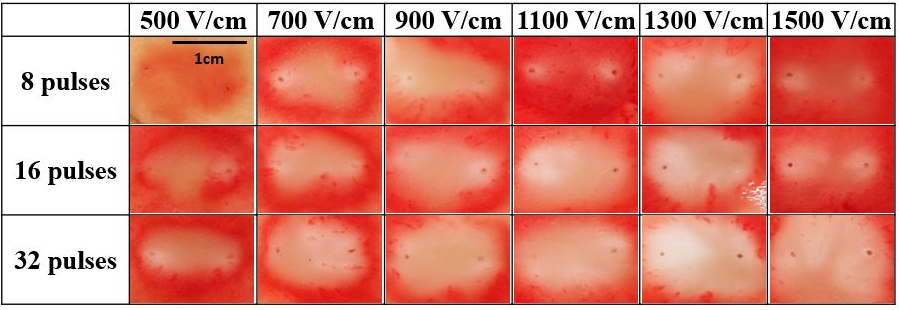


Supplementary Table 2. TTC staining on potato tissue 3 h posttreatment at various pulse delay with 1000 V/cm pulse voltage, 36 pulse number


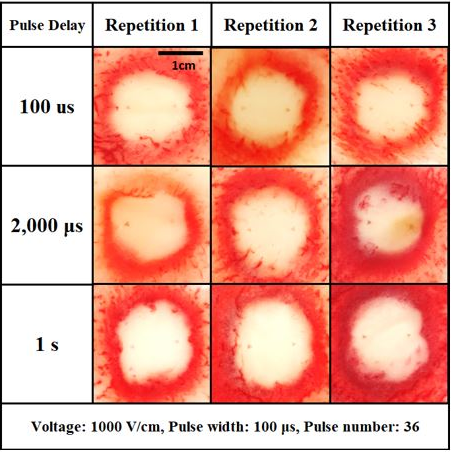


Supplementary Table 3. Conductivity change rate before and after IRE treatment on potato at various voltage conditions depending on the frequency (%)

|  | 10 Hz | 100 Hz | 1,000 Hz | 10,000 Hz | 100,000 Hz | 1,000,000 Hz | 10,000,000 Hz |
| --- | --- | --- | --- | --- | --- | --- | --- |
| 300 V/cm | 4.1 | -1.5 | 0.8 | 2.6 | 18.6 | 8.1 | -1.0 |
| 500 V/cm | 20.4 | 13.9 | 16.2 | 17.0 | 34.2 | 11.1 | -1.5 |
| 700 V/cm | 27.3 | 23.3 | 28.6 | 32.2 | 48.2 | 11.9 | -1.7 |
| 900 V/cm | 31.9 | 28.7 | 39.5 | 51.5 | 60.9 | 8.8 | -1.3 |
| 1100 V/cm | 83.1 | 75.3 | 78.7 | 76.9 | 85.5 | 21.0 | 0.5 |

Supplementary Figure 1. TEM analysis of non-pulsed perimedullar zone with evident electron-dense line (arrows) along the cell wall (CW). **(A)** The well-preserved plasma membrane overall attached to the cell wall in the perimedullar zones of the tuber in the non-pulsed perimedullar zone. Organelles, such as Golgi bodies (G) and mitochondria (M), were commonly observed in the cytoplasm. **(B)** The cisternae, lumens, and secretory vesicles could be discerned from the Golgi body. **(C)** Magnified views showed double membranes and cristae of the mitochondria. **(D)** Complex plasmodesmata (Pd) were often seen between two adjoining parenchyma cells (P).


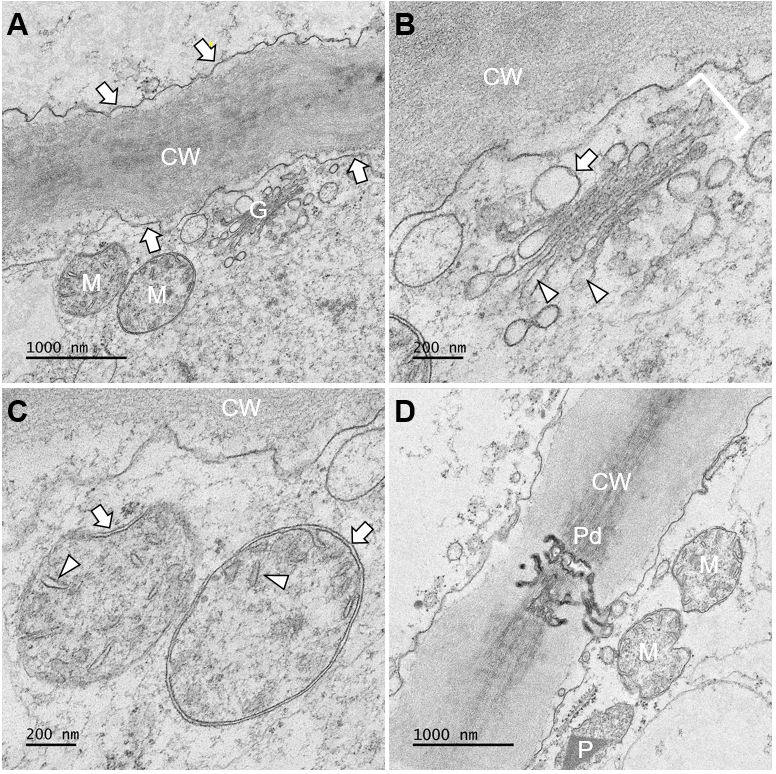


Supplementary Figure 2. TEM analysis of pulsed TTC-unstained medullar zone with no electron-dense line along with the CW. **(A)** The cytoplasmic disruption occurred in the IRE inner medullar zone. **(B)** Magnified views showed the pulsed cytoplasm partially contained electron-dense vesicles with various diameters. The absence of an electron-dense continuous plasma membrane was commonly observed along the cell wall in the magnified views. **(C)** No distinct organelles were found in the cells. **(D)** Magnified views showed the disrupted cytoplasm and plasma membrane detached (to ~1 μm) from the cell wall.


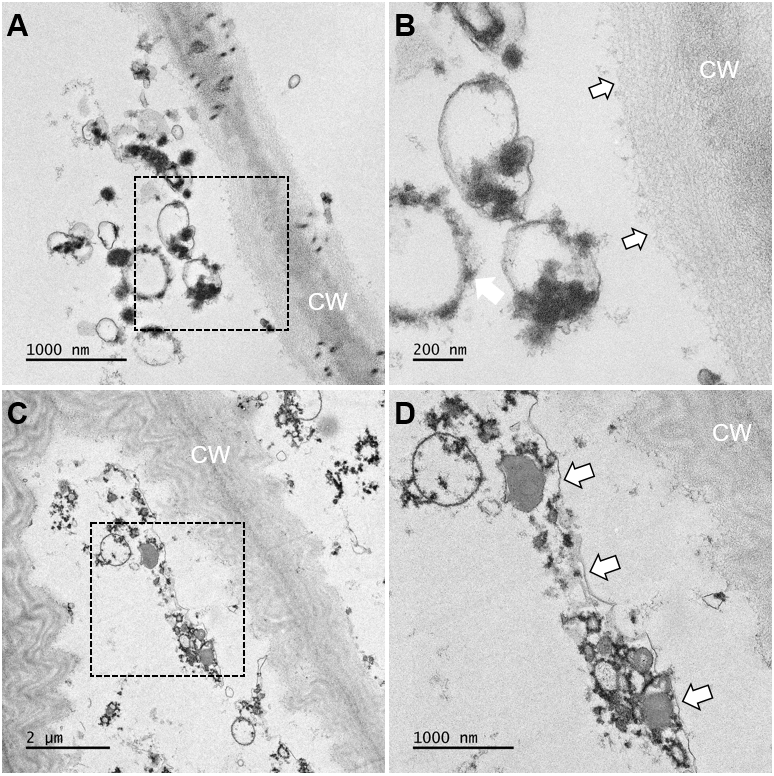


Supplementary Figure 3. TEM analysis of pulsed TTC-stained deep red inner medullar zone with partially continuous electron-dense lines along with the CW. **(A)** Partially continuous electron-dense plasma membranes were commonly found in the TTC-stained deep red inner medullar zone. **(B)** Magnified views showed the dotted electron-dense remains and detachment of the plasma membrane (to ~500 nm) from the cell wall. **(C)** Partially disrupted organelles, including Golgi bodies, were found near the plasma membrane in the cells. **(D)** Small vesicles, ~100 nm or less in diameter, were observed between the plasma membrane and cell wall. Vesicular bodies (VB) appeared to fuse with the plasma membrane.


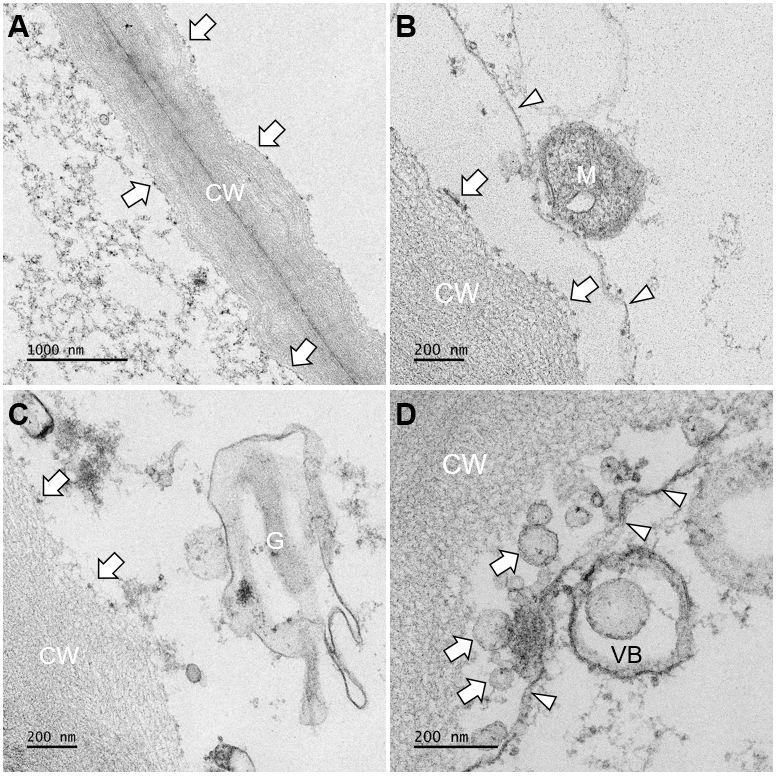

Supplement: Supplementary file 1 — Supplementary Information. [file 41598_2021_99987_MOESM1_ESM.docx]
